# Intranasal administration of *Ganoderma lucidum*-derived exosome-like nanovesicles ameliorates cognitive impairment by reducing inflammation in a mouse model of Alzheimer’s disease

**DOI:** 10.3389/fphar.2025.1572771

**Published:** 2025-07-04

**Authors:** Xue Mi, Xinglin Ruan, Renyi Lin, Shuxin Huang, Ping Cai, Xiaochun Chen, Jiangfeng Liao, Xiaoman Dai

**Affiliations:** ^1^ Public Technology Service Center, Fujian Medical University, Fuzhou, China; ^2^ Fujian Key Laboratory of Molecular Neurology, Institute of Neuroscience, Fujian Medical University, Fuzhou, China; ^3^ Department of Neurology and Geriatrics, Fujian Institute of Geriatrics, Fujian Medical University Union Hospital, Fuzhou, China; ^4^ School of Pharmacy, Fujian Medical University, Fuzhou, China; ^5^ School of Public Health, Fujian Medical University, Fuzhou, China; ^6^ Department of Neurology, First Affiliated Hospital of Fujian Medical University, Fuzhou, China

**Keywords:** *Ganoderma. lucidum* (*G. lucidum*)-derived exosome-like nanovesicles, cognitive impairment, inflammatory response, Alzheimer’s disease, intranasal administration

## Abstract

**Background/Objectives:**

Although Alzheimer’s disease (AD) is the most prevalent dementia in late life, with amyloid beta (Aβ) deposition and neuroinflammation are recognized among its primary pathological features. Currently, there is currently still a lack of effective therapeutic drugs for AD. *Ganoderma lucidum* (*G. lucidum*) is abundant in active ingredients that harbor anti-inflammatory properties in both central nervous system and the periphery. We attempted to determine whether *G. lucidum* contained exosome-like nanovesicles (GLENVs) and whether these GLENVs can alleviate cognitive impairment.

**Methods:**

We extracted GLENVs by the differential ultracentrifugation method and identified the components by liquid chromatography-mass spectrometry (LC-MS). The 5×FAD mice underwent a 3-month intranasal administration of GLENVs and their behavioral and pathological changes were evaluated.

**Results:**

GLENVs were successfully extracted and identified to contain multiple ganoderic acids; intranasal administration allowed GLENVs to penetrate the blood-brain barrier to exert their effects directly. The 3-month GLENVs treatment effectively ameliorated the impairment in the memory and learning of the 5×FAD mice. The GLENVs treatment also reduced Aβ deposition in the cortex and hippocampus of 5×FAD mice, overactivated microglia, reactive astrocytes, and pro-inflammatory factors, and inhibited the Janus kinase 2 (JAK2)/Signal transducer and activator of transcription 3 (STAT3) signaling pathway. Moreover, GLENVs exerted no adverse effects on liver and kidney function.

**Conclusion:**

GLENVs may be a promising candidate for AD treatment.

## 1 Introduction

With the extending life expectancy, aging-related diseases such as dementia have become increasingly prevalent, with Alzheimer’s disease (AD) as the most common form of dementia in late life by far. By 2030, about 74.7 million people will be afflicted with dementia and the cost of caring may approximate to $2 trillion. Without effective therapies, the number of people with dementia is expected to reach 131.5 million by 2050 (Cummings et al., 2016). Currently, there are only seven FDA-approved drugs for the clinical treatments of AD. Among them, the three cholinesterase inhibitors and the NMDA antagonist memantine have demonstrated efficacy and sufficient safety for a wide clinical application. However, these four agents can only provide symptomatic relief and temporary improvement in memory and thinking. They cannot slow down or reverse the progression of the diseases (Athar et al., 2021; Schneider and Sano, 2009). Of the FDA-approved monoclonal antibody drugs for AD, namely, aducanumab (Walsh et al., 2021), lecanemab (van Dyck et al., 2023) and donanemab (Wang et al., 2025), have been found to induce significant adverse reactions in some patients, though beneficial in reducing amyloid plaques in patients’ brains. Therefore, it is of important clinical value and social significance to explore more effective and safer drugs for the prevention and treatment of AD.

Currently, studies have documented inflammatory changes and overactivation of microglia within the brains of both AD animal models and patients (Onyango et al., 2021; Andronie-Cioara et al., 2023) and amyloid beta (Aβ) deposition as a crucial contributing factor for neuroinflammation (Heneka et al., 2015). Thus,in AD brain, the continuously-overactivated microglia can trigger the release of pro-inflammatory factors, which in turn promote neuroinflammation, aggravating Aβ deposition and neuronal death (Khan et al., 2020). Several investigations have revealed that a long-term administration of nonsteroidal anti-inflammatory drugs may reduce the risk for AD (McGeer et al., 1996). Together, these findings indicate a close association between neuroinflammation and AD pathogenesis, which suggests that reducing neuroinflammation might present a potential approach to AD treatment.

Medicinally, *Ganoderma Lucidum* (*G. lucidum*), a medicinal mushroom, has been traditionally used in the East Asia for its therapeutic properties and its potential to promote longevity (Wang et al., 2017; Ahmad, 2018). It contains numerous biological active constituents that can confer neuroprotective effects, such as alleviating neuroinflammation, through diverse mechanisms (Ahmad et al., 2021). Of the biological active constituents in *G. lucidum*, *Ganoderma lucidum* triterpenoids (GLTs) and polysaccharides (GLPs) are the principal bioactive and medicative components (Galappaththi et al., 2022; Seweryn et al., 2021). Studies have indicated that GLTs and GLPs can improve cognitive impairments in AD mice (Huang et al., 2017; Qi et al., 2021). Nevertheless, relatively few reports have explored the effects of *G. lucidum* on clinical patients, which is probably due to the difficulties that the biological active constituents of *G. lucidum* encounter in penetrating the blood-brain barrier (BBB). In addition, the extraction and purification of the active components of *G. lucidum* demand a substantial number of organic solvents, which increases not only the financial costs but also the toxicity. Consequently, it is essential to explore novel dosage forms of *G. lucidum* that boast a reasonable safety and affordability and an ease in BBB crossing.

Currently, extensive attention has been drawn to the nanoparticle-based medicinal delivery system (Mondal et al., 2023). As a type of extracellular vesicle, exosomes are secreted by a multitude of cell types, such as mesenchymal stem cells (MSCs). Exosomes feature a bilayer membrane structure and a diameter ranging from 40 to 160 nm. They are replete with a wealth of functional components, including DNA, RNA, lipids, metabolites, and proteins (Kalluri and LeBleu, 2020). Some studies have documented core AD biomarkers in blood exosomes during AD diagnosis, which indicates that exosomes can also penetrate the BBB (Hu et al., 2024). Other studies have synthesized exosome-liposome hybrid nanovesicles to modulate microglial dysfunction and Aβ anabolism, thus rescuing the cognitive function of the APP/PS1 mouse model (Jiang et al., 2024), suggesting the potential clinical applications of these exosome-related nanovesicles for AD treatment. Meanwhile, exosome-like nanovesicles are also present in the natural resources used in Traditional Chinese Medicine. Available studies have documented that naturally-derived exosome-like nanovesicles contain not only a substantial quantity of proteins and RNAs but also the inherent active ingredients (Kim et al., 2023). However, despite the potent pharmacological activities of the *G. lucidum* components and the possibilty of isolating exosome-like nanovesicles from a variety of macrofungus (Liu et al., 2020), it remains unexplored whether exosome-like nanovesicles can be extracted from *G. lucidum* and whether the *G. lucidum*-derived exosome-like nanovesicles (GLENVs) encompass diverse bioactive compounds and will confer the desired pharmacological effects. This study tackled this very issue and explored the impact of GLENVs on the cognitive function and concomitant pathological alterations in 5×FAD mice. We also investigated the Aβ deposition and neuroinflammation in the 5×FAD mice after GLENVs treatment. The findings may highlight the value of GLENVs as preventive and therapeutic drugs for AD treatment regimens.

## 2 Materials and methods

### 2.1 Experimental protocol

The overall study protocol was briefly explained as follows. The GLENVs were obtained by differential ultracentrifugation method and used for follow-up experiments after characterization. All the animals were housed under standard conditions. GLENVs or vehicle treatment was initiated for all mice at 5 months of age and continued for 3 months. Behavioral tests were performed on the day after the closure of medication administration. After the behavioral tests, all the mice were euthanized *via* a complete isoflurane anesthesia and the peripheral blood samples and brains were collected for analyses.

### 2.2 Animals

The 5×FAD mice, a mouse model co-expressing five familial AD mutations on the human amyloid precursor protein, were procured from Jackson Laboratory (Stock no. 034848-JAX; Bar Harbor, ME, United States). The control mice consisted of littermate of wild-type (WT) mice. The experimental group was composed of an equal number of both male and female mice. All the animals were raised under standard housing conditions (no more than six mice per cage; on a 12-h light/dark cycle, with lights turned on at 7: 00 a.m.; humidity: 50% ± 10%; and temperature: 22°C ± 1°C), with water and food accessed freely. All animal-related experimental designs were reviewed by the Ethical Committee of Institutional Animal Care and Use of Fujian Medical University (IACUC FJMU 2024-0406). The experimental procedures observed the European Community Guidelines for the Care and Use of Experimental Animals (Directive 2010/63/EU).

### 2.3 Isolation and characterization of GLENVs

The fruiting bodies of *G. lucidum* were obtained from Mount Wuyi *G. lucidum* Planting Base (Xianzhi Technology (Fujian) Co., Ltd.), and identified as *G. lucidum* (Leyss. ex Fr.) Karst. by the Institute of Microbiology, Chinese Academy of Sciences. A voucher specimen (23609) was preserved in our laboratory

The exosome-like nanovesicles were extracted from *G. lucidum* and characterized, following a previous method, with modification (Liu et al., 2020). The entire *G. lucidum* (cap and stem) was meticulously cleaned and minced. Subsequently, the minced *G. lucidum* (100 g) was immersed into a cold phosphate buffer solution (PBS, 1 L) and extracted with an electric juicer to obtain the *G. lucidum* juice by filtration with a muslin cloth. The *G. lucidum* juice was then successively centrifuged at 1,000 × g for 10 min, 2,000 × g for 20 min, 10,000 × g for 30 min to eliminate large fibers. Afterwards, the supernatant was ultracentrifugated at 100,000 × g for 1 h. The obtained precipitate was subjected to a resuspension in PBS and ultracentrifugation (100,000 × g for 1 h). Finally, the precipitate, designated as GLENVs, was resuspended in PBS (12 mL) and stored at −80°C until use. The GLENVs used for drug administration were filtered through an organic filter membrane (0.22 μm). For the characterization of GLENVs, images of GLENVs were captured by transmission electron microscopy (TEM). The particle size and concentration were measured by nanoparticle tracking analysis (NTA) with ZetaView^®^ (Particle Metrix, Germany) and the corresponding software ZetaView v8.05.14. Proteins were extracted from GLENVs with a cold RIPA buffer (9806, CST; Danvers, MA, United States) consisting of phosphatase inhibitors, protease, and PMSF (5870, 5871, 8553, CST; Danvers, MA, United States). The protein level was measured with an Enhanced BCA Protein Assay Kit (P0010, Beyotime, Shanghai, China).

### 2.4 TEM analysis of GLENVs

A drop of GLENVs sample was carefully deposited onto the surface of a formvar-coated copper grid. Subsequently, 2% uranyl acetate was added for a 5-min staining. The whole procedure was performed in dark. Afterwards, the sample was dried for subsequent imaging. The image was captured under a Tecnai G2 TEM.

### 2.5 LC-MS/MS analysis

The concentration of the reported active compounds of *G. lucidum* within the GLENVs was quantified by triple quadrupole liquid chromatography-mass spectrometry (LC-QqQ MS/MS) in accordance with the reported method (Lin et al., 2024).

#### 2.5.1 Preparation of standard solutions

The reference standards of Ganoderic acids A, B, D, F, G, I, Lucidenic acid A, and Ganoderenic acid D were precisely weighed and dissolved in methanol. The concentration of each standard stock solution was set at 1,000 μg/mL and stored at −20°C. The composite stock solution was prepared by mixing the eight analytes in proper proportions and subsequently diluted with methanol to prepare a series of different concentrations, thereby obtaining the working solution. These working solutions were stored at 4°C and filtered through syringe filters (0.22 μm) before injection into the LC–MS/MS system.

#### 2.5.2 Sample preparation

For the GLENVs sample, 30 µL of GLENVs was thoroughly mixed with 150 µL of methanol by vortexing for 5 minutes. Subsequently, the mixture was centrifuged at 12,000 rpm at 4°C for 10 minutes. Thereafter, the supernatant was carefully removed into a new 1.5 mL centrifuge tube and subjected to a second centrifugation at 12,000 rpm and 4°C for another 10 minutes. The resulting supernatant was then collected in a liquid-phase chromatography vial for subsequent analysis.

The brain samples were next utilized to identify the components that had penetrated the brain. The mouse olfactory bulb, cortex, hippocampus and hypothalamus tissue were dissected on ice and homogenized in PBS. Subsequently, the tissue homogenate was thoroughly mixed with five volumes of methanol, sonicated for 10 minutes, and then vortexed for 5 minutes. This mixture was next centrifuged at 4°C and 12,000 rpm for 10 minutes. Afterwards, the supernatant was transferred into a new 1.5 mL centrifuge tube and recentrifuged. The final supernatant was collected, dried, redissolved in methanol, and transferred in vials for liquid-phase chromatography before analysis.

#### 2.5.3 Chromatographic conditions

The samples were examined on an UPLC–MS/MS 8040 system provided by Shimadzu, which comprised an LC-20AD binary pump, a SIL-20AC autosampler, an FCV-20A controller, and a CTO-20A column oven. The chromatographic separation was proceeded with a Welch Ultimate XB-C18 (2.1 mm × 50 mm, 3 μm) along with a guard column (2.1 mm × 5 mm, 3 μm). The mobile phase consisted of water with 0.1% (*v/v*) formic acid (A) and methyl alcohol (B). The flow rate was maintained at 0.2 mL/min. The needle washing liquid was a mixture of 50% methanol and water, and the column oven temperature was maintained at 30°C. The injection volume was set at 2 µL. The following chromatographic gradient was utilized in this experiment: from 0 to 0.5 min, 90% A; from 0.5 to 2.5 min, a linear change from 90% A to 10% A; from 2.5 to 8 min, 10% A; from 8 to 20 min, 90% A.

#### 2.5.4 Mass spectrometry conditions

Mass spectrometry was performed with a Shimadzu LC–MS/MS 8040 triple quadrupole mass spectrometer with an electrospray ionization (ESI) interface. The detection of analytes in the negative ion mode was performed by multiple reaction monitoring (MRM). The specific conditions of the ESI source included: the heat block temperature was maintained at 400°C; the desolvation line (DL) temperature was set at 250°C; the flow rate of the nebulizing gas (N_2_) was 3 L/min; and the flow rate of the drying gas (N_2_) was 15 L/min.

#### 2.5.5 Sample determination

The compounds were quantified and identified according to the MRM transition and retention time of each compound. The response signals of the compounds were detected by scanning in the negative ion mode. According to the detected signal, the one with a relatively higher signal value and more distinct mass spectra was selected for quantitative analysis. The concentrations of the eight selected analytes were calculated from the respective external standard calibration curves.

### 2.6 Drug administration

Mice were reared until 5 months of age and then randomly assigned to four groups (10–15 mice per group): the WT + PBS group; the Control + GLENVs group; the 5×FAD + PBS group; and the 5×FAD + GLENVs group. The Control + GLENVs and 5×FAD + GLENVs groups received GLENVs *via* nasal drops at a dosage of 1 μg/μL, to a total of 20 μL per day, for three consecutive months. The WT + PBS and 5×FAD + PBS groups received PBS to simulate nasal drop stress and rule out the influence of vehicle.

### 2.7 Behavioral tests

All experiments were performed in a double-blind manner and the sequence of testing was counterbalanced to minimize potential biases. Before each test, all mice were placed in the behavioral testing room to acclimate to the testing environment for at least 1 h. The behavioral tests were conducted separately, with an interval of 1–2 days in between. After each test, the testing equipment was meticulously wiped with 75% ethanol to remove any residual odors that might influence the performance of the other mice. Mice with visual impairments, abnormal behaviors, or an inability to swim were excluded from the final statistical analysis.

#### 2.7.1 Morris water maze (MWM) test

The MWM test was adopted to evaluate the spatial learning and memory of mice, following a previously described method with slight modification (Lin et al., 2023). Briefly, the maze comprised a dark circular pool (diameter: 120 cm), with a platform (diameter: 7 cm) located in the center of the southeast corner of the pool. The pool was filled with opaque water (depth: 35 cm) to a depth of 1.5 cm higher than the height of the platform. The water temperature was maintained at 22°C ± 2°C. Simple patterns of various colors and geometric shapes were affixed to the inner walls of the pool, serving as extra-maze cues. The experimental training period lasted for five consecutive days, during which the mice were trained four times daily. During each trial, the mice were placed into the water, facing the wall of the pool, from a different location according to the semirandom sequence distribution. They were given 60 s to freely explore the pool to locate the platform. Those that failed to locate the platform within 60 s were guided to the platform manually and allowed to stay on the platform for 20 s. On the sixth day, a memory retention test was initiated with the platform removed. Each mouse was permitted to explore the pool for 60 s. The swimming performance of the mice were automatically recorded and analyzed with an animal behavior analysis software (ANY-MAZE, Stoelting, United States).

#### 2.7.2 Open field test (OFT)

The OFT was proceeded as mentioned previously, with slight modification (Mi et al., 2022). This test was performed in a dimly-lighted, low-noise environment. The testing apparatus comprised an opaque plastic open field arena in a Plexiglas box (*L* × *W* × *H*: 44 × 44 × 40 cm). The arena was divided into a central square (22 × 22 cm), four corner areas (11 × 11 cm each), and other surrounding zones. Each animal was placed in one corner of the arena and allowed to explore the arena freely for 10 minutes. The time spent and the locomotor activity in the central and corner zones were recorded with the Shanghai Xinsoft Behavioral Software.

#### 2.7.3 Y-maze test

The Y-maze test was utilized to assess spatial memory, as described in previous studies (Golderman et al., 2022). The Y-shaped apertures were composed of three arms (one start arm and two objective arms) (*L* × *W* × H: 30 × 6 × 30 cm for each arm). Spatial cues were provided in the form of a square stripe label positioned at the entrance of the familiar arm and a triangle at that of the novel arm. The animals were initially placed in one arm of the Y-maze (the start arm), while one of the other two arms (the novel arm) was blocked. They were permitted to explore the start arm and the remaining accessible arm (the familiar arm) for 5 minutes. Subsequently, they were returned to their home cage for a 1-h break. During the test, the novel arm was opened and the test mice were reintroduced into the start arm and allowed to explore all the three arms freely for 5 minutes. The time spent in each arm of the maze and the entries into the novel arm were recorded. A preference index was calculated to distinguish between novelty and familiarity preferences: Preference index = the time spent in the novel arm/the total exploration time of the novel and the familiar arms × 100%.

#### 2.7.4 Novel object preference (NOP) and novel location preference (NLP) test

The NOP test was adopted to measure non-spatial memory in the mice, following the method reported in the previous review (Chao et al., 2022). The test consisted of a habituation, an acquisition and a test trial, with temporal intervals in between. In the habituation phase, the animals were individually given 10 minutes to acclimate to an open field arena (42 × 42 × 42 cm), 24 h before testing. During the acquisition phase, two identical novel objects were placed at the two adjacent corners of the arena, and the mice were then allowed to explore the arena freely for 10 minutes. The test trial was proceeded 1 hour after the acquisition phase, during which one of the objects in the arena was replaced with a novel object of a different color and shape. The familiar and novel objects were positioned in the same locations as in the acquisition trial, ensuring that their spatial locations remained unchanged. The exploratory behavior was once again assessed for 10 minutes. Typically, rodents exhibited a greater tendency to explore the novel object when compared with the familiar one, which indicates recognition memory for the previously-explored object. All objects were cleansed thoroughly with 75% ethanol between sessions to eliminate any residual olfactory cues that might be recognized. In all trials, the objects used were ordinary items of similar size but with distinct shape texture and color, clearly distinguishable from the background. Exploration behavior was defined as the actions of mice touching the object with the nose and/or forepaws or directing their nose toward the object within 2 cm.

The protocol of the NLP test resembled that of the NOP test, with the exception that one of the familiar objects in the test trial was relocated to a novel position. Since the objects themselves were unreplaced, “novelty” in this case was the spatial alteration rather than the object itself. The preference for visual novelty was calculated using the following formula: (the exploration time dedicated to the novel object or location/the total exploration time of the novel + familiar objects or location) × 100%.

### 2.8 Enzyme-linked immunosorbent assay (ELISA)

Brain samples were obtained from 5×FAD and WT mice and then homogenized with PBS. The levels of interleukin-1β (IL-1β, RX203063M)), interleukin-6 (IL-6, RX203049M), and tumor necrosis factor-α (TNFα, RX202412M) in the brains were analyzed with a commercial kit (Ruixinbio, Quanzhou, China) in accordance with the instructions from the manufacturer.

### 2.9 RNA extraction and quantitative real-time PCR (qPCR)

qPCR was proceeded as previously established (Mi et al., 2022). The brains of the test mice were harvested after a cardiac perfusion with 0.01 M PBS, placed in precooled PBS, and then sliced into coronal sections (1 mm thick). The cortex and hippocampus were dissected separately and rapidly frozen in liquid nitrogen and immediately stored at −80°C until analysis. Total RNA was extracted with a TriZol reagent (R401-01, Vazyme, NanJing, China) and the reverse transcription was synthesized with HiScript II Q RT SuperMix for qPCR (R223-01, Vazyme, NanJing, China) in accordance with standard protocols. The cDNAs of IL-6, IL-1β, and TNF-α were amplified by real-time quantitative PCR (RT-qPCR) using Hieff UNICON^®^ advanced qPCR SYBR Master (11185ES08, Yeasen, Shanghai, China). Mouse GAPDH was employed as internal control. The relative gene expression was determined by the 2^−ΔΔCT^ method. The primers for RT-qPCR included: For GAPDH, Forward primer (F), 5′-AGG​TCG​GTG​TGA​ACG​GAT​TTG-3′, Reverse primer (R), 5′-TGT​AGA​CCA​TGT​AGT​TGA​GGT​C A-3′; For IL-1β: Forward primer (F), 5′-TCG​CAG​CAG​CAC​ATC​AAC​AAG​AG-3′, Reverse primer (R), 5′-AGG​TCC​ACG​GGA​AAG​ACA​CAG​G-3′; For IL-6: Forward primer (F), 5′-CTCCCAACA GACCTGTCTATAC-3′, Reverse primer (R), 5′-CCA​TTG​CAC​AAC​TCT​TTT​CTC​A-3′; For TNF-α: Forward primer (F), 5′-ACT​GGC​AGA​AGA​GGC​ACT​CC-3′, Reverse primer (R), 5′-GCCACAAG CAGGAATGAGAA-3′.

### 2.10 Western blot analysis

The levels of proteins were analyzed by standard Western blot assays as established previously (Zhuang et al., 2024). Briefly, the total protein of brain tissue samples was extracted with cold RIPA protein extraction reagent (P0013C, Beyotime, Shanghai, China) supplemented with protease and phosphatase inhibitors (HY-K0010, HY-K0022; MCE, New Jersey, United States) and PMSF (ST506, Beyotime, Shanghai, China) on ice for 30 min. Subsequently, the samples were centrifuged at 12,000 g and 4°C for 20 min. The supernatant was then collected and quantified with the Enhanced BCA Protein Assay Kit (P0010, Beyotime, Shanghai, China) in accordance with the instructions from the manufacturer. Each sample containing 30 μg of protein extract was subjected to SDS-PAGE, with 4%–12% Bis-Tris SurePAGE gels (36255ES10, Yeasen, Shanghai, China). After electrophoresis, the resolved sample proteins were transblotted onto PVDF membranes (0.2 μm; IPVH00010, Immobilon-P, Ireland). Blocking was carried out with 5% bovine serum albumin (BSA; 0332, VWR, Radnor, Pennsylvania, United States) at room temperature for 1 hour. Then, the membranes were incubated at 4°C overnight in specific primary antibodies (Abcam, Cambridge, UK: β-actin, 1:10,000, ab8226; Iba1, 1:1,000, ab5076; APP, 1:1,000, ab32136. Biolegend, San Diego, California, United States: Aβ 6E10, 1:2,000, 803,004. Affinity Biosciences, Liyang, China: JAK2, 1:1,000, AF6022; pJAK2-Tyr1007, 1:1,000, AF3024. CST, Danvers, United States: STAT3, 1:1,000, #9132; p-STAT3-Tyr705, 1:1,000, #9131; GFAP, 1:2,000, #3670; Caspase-8, 1:1,000, #9746; BACE, 1:1,000, #5606; NRF2, 1:1,000, #12721. Proteintech, Wuhan, China: ZBP1, 1:1,000, 22803-1-AP). The membranes were subsequently rinsed with TBST three times (10 min each) and incubated in a secondary antibody (1:10,000; C31430, C31460, A15999, Invitrogen™, Carlsbad, California, United States) at room temperature for 1 hour. Then, the membranes were rinsed in TBST for three times (10 min each) and detected with the Enhanced ECL Chemiluminescent Substrate Kit (36222ES60, Yeasen, Shanghai, China). Grayscale analysis was conducted with the NIH ImageJ software.

### 2.11 Thioflavin S (TS) staining

Ab plaques were labeled by TS staining. This experiment was conducted as demonstrated previously, with modifications (Pan et al., 2022). Briefly, brain sections were stained with 0.002% TS (T1892-25G, Sigma-Aldrich, Japan) in 50% ethanol in the dark for 8 min, followed by two rinses with 50% ethanol and three rinses with PBS. Thereafter, the brain sections were mounted with an anti-quenching mounted medium (S36968, Invitrogen, Carlsbad, California, United States) and observed under an APX100 All-in-One microscope (Olympus, Japan).

### 2.12 Assays of serum biochemical parameters

The measurements of aspartate aminotransferase (AST), serum alanine aminotransferase (ALT), blood urea nitrogen, creatinine, and uric Acid were conducted following the instructions of the detection kits (Ruixinbio, Quanzhou, China), respectively.

### 2.13 Statistical analysis

Data were reported as mean ± SEM and analyzed with GraphPad Prism 9 software. Inter-group differences and statistical significance between four groups were evaluated by two-way analysis of variance (ANOVA), with the Turkey test for the *post hoc* multiple-comparisons, and the comparison between two groups by unpaired t-test. A p value of <0.05 was deemed as statistically significant.

## 3 Results

### 3.1 Extraction of exosome-like nanovesicles s from *G. lucidum*

**FIGURE 1:**
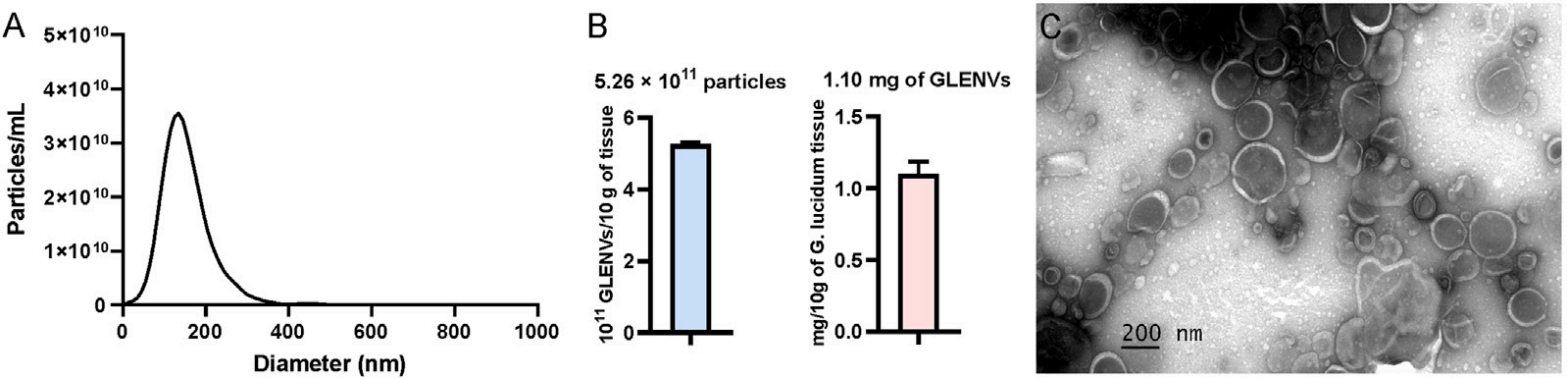
The characterization of *G. lucidum*-derived exosome-like nanovesicles (GLENVs). **(A)** Size distribution of the GLENVs with a peak of 130.6 nm. **(B)** Number of GLENVs particles (left) and protein concentration (right) in per 10 g of *G. lucidum* tissue. **(C)** Morphology of GLENVs by transmission electron microscopy (TEM).

Dietary exosome-like nanovesicles have been extracted from several commonly consumed mushrooms (Liu et al., 2020). To ascertain whether *G. lucidum* contains exosome-like nanovesicles, the fruiting bodies of *G. lucidum* were isolated by a differential ultracentrifugation method and then detected, which indicated that exosome-like nanovesicles were successfully obtained from *G. lucidum* and thus designated as GLENVs. NTA was employed to investigate the size distribution range of GLENVs, which revealed that the peak value of GLENVs size was approximately 130.2 nm (Figure 1A). The concentration of GLENVs, calculated by the Zeta View and after conversion, was around 5.26 × 10^11^ particles per 10 g fruiting bodies of *G. lucidum* (Figure 1B). TEM analysis demonstrated that the GLENVs featured discrete nanoparticles with a typical cup-shaped structure (Figure 1C). Meanwhile, the concentration approximated to 1.10 mg/mL by the Bradford assay (Figure 1B).

### 3.2 Quantitative analysis of GLTs in GLENVs

GLTs are the representative bioactive ingredient of *G. lucidum*. To ascertain whether GLENVs contained any GLTs, eight classical active components, including ganodermic acids A, B, D, F, G, I, Lucidenic acid A, and ganodermic acid D, were quantified by LC-MS/MS. The MRM transition and retention time of each compound are presented in Table 1. Representative chromatograms of the extracted ions in standard solutions and samples are exhibited in Supplementary Figure S1. The content of compounds in four different batches of GLENVs was determined, with the results illustrated in Table 2. In this experiment, all the selected compounds were detected in all four batches and quantified by the external standard method. The standard curve, linear regression equation, and correlation coefficient are shown in Supplementary Figure S2. Among the four batches, ganoderic acid A reported the highest content, with an average of 3.449 ng per Gram of fruiting bodies of *G. lucidum*. The findings indicate that these tested compounds have significantly contributed to the pharmacological effect of GLENVs.

**TABLE 1:** Identification and quantitative information of classical active components of *G. lucidum* in GLENVs.

Components	CAS	Formula	MW	Mode	RT (min)	Parent-ion	Fragment-ion	CE
Ganoderic acid A	81907-61-1	C_30_H_44_O_7_	516.666	[M-H]^-^	7.650	515.35	300.25	32
Ganoderic acid B	81907-61-1	C_30_H_44_O_7_	516.666	[M-H]^-^	7.435	515.35	249.30	38
Ganoderic acid D	108340-60-9	C_30_H_42_O_7_	514.650	[M-H]^-^	7.621	513.30	149.20	45
Ganoderic acid F	98665-15-7	C_32_H_42_O_9_	570.670	[M-H]^-^	7.593	569.30	551.20	16
Ganoderic acid G	98665-22-6	C_30_H_44_O_8_	532.666	[M-H]^-^	7.444	531.40	265.25	37
Ganoderic acid I	98665-20-4	C_30_H_44_O_8_	532.670	[M-H]^-^	7.246	531.30	129.20	21
Lucidenic acid A	95311-94-7	C_32_H_42_O_9_	458.587	[M-H]^-^	7.693	457.30	149.20	40
Ganoderenic acid D	100665-43-8	C_30_H_40_O_7_	512.634	[M-H]^-^	7.563	511.30	493.20	17

**TABLE 2:** Detection of the eight active components in GLENVs (ng/g).

Components	The first batch	The second batch	The third batch	The fourth batch
Ganoderic acid A	3,125.79	3,469.99	3,490.27	3,709.92
Ganoderic acid B	185.63	180.88	180.40	157.72
Ganoderic acid D	605.49	696.06	603.28	760.56
Ganoderic acid F	370.33	410.29	359.27	425.43
Ganoderic acid G	251.12	238.95	227.75	193.44
Ganoderic acid I	162.96	173.65	158.08	191.49
Lucidenic acid A	52.70	55.17	55.25	44.48
Ganoderenic acid D	201.05	213.58	225.14	256.82

### 3.3 Detection of ganoderic acid A, D, and F in the brain after intranasal administration of GLENVs

To assess brain penetration of GLENV, the levels of ganoderic acids A, D and F in the olfactory bulb, cortex, hippocampus, hypothalamus of mice were quantified by LC-MS/MS 2 hours after the intranasal administration of 20 μL GLENVs. As detailed in Table 3, ganoderic acid A, D and F were detected in all three mice, despite the noticeable difference, the measured results in the three mice indicated that GLENVs successfully traversed across the BBB and entered the brain.

**TABLE 3:** Detection of Ganoderic acid A, D and F in the brain after intranasal administration of GLENVs (n = 3).

Brain tissues	Ganoderic acid A (ng/mg)	Ganoderic acid D (ng/mg)	Ganoderic acid F (ng/mg)
olfactory bulb	22.90 ± 1.46	3.58 ± 0.18	2.75 ± 0.23
cortex	4.72 ± 0.67	0.58 ± 0.08	1.54 ± 0.19
hippocampus	9.03 ± 0.42	1.78 ± 0.09	1.10 ± 0.05
hypothalamus	7.20 ± 2.24	1.47 ± 0.53	3.19 ± 1.04

### 3.4 Three-month treatment with GLENVs ameliorates cognitive dysfunction in learning and spatial memory impairment in the 5×FAD mice

**FIGURE 2:**
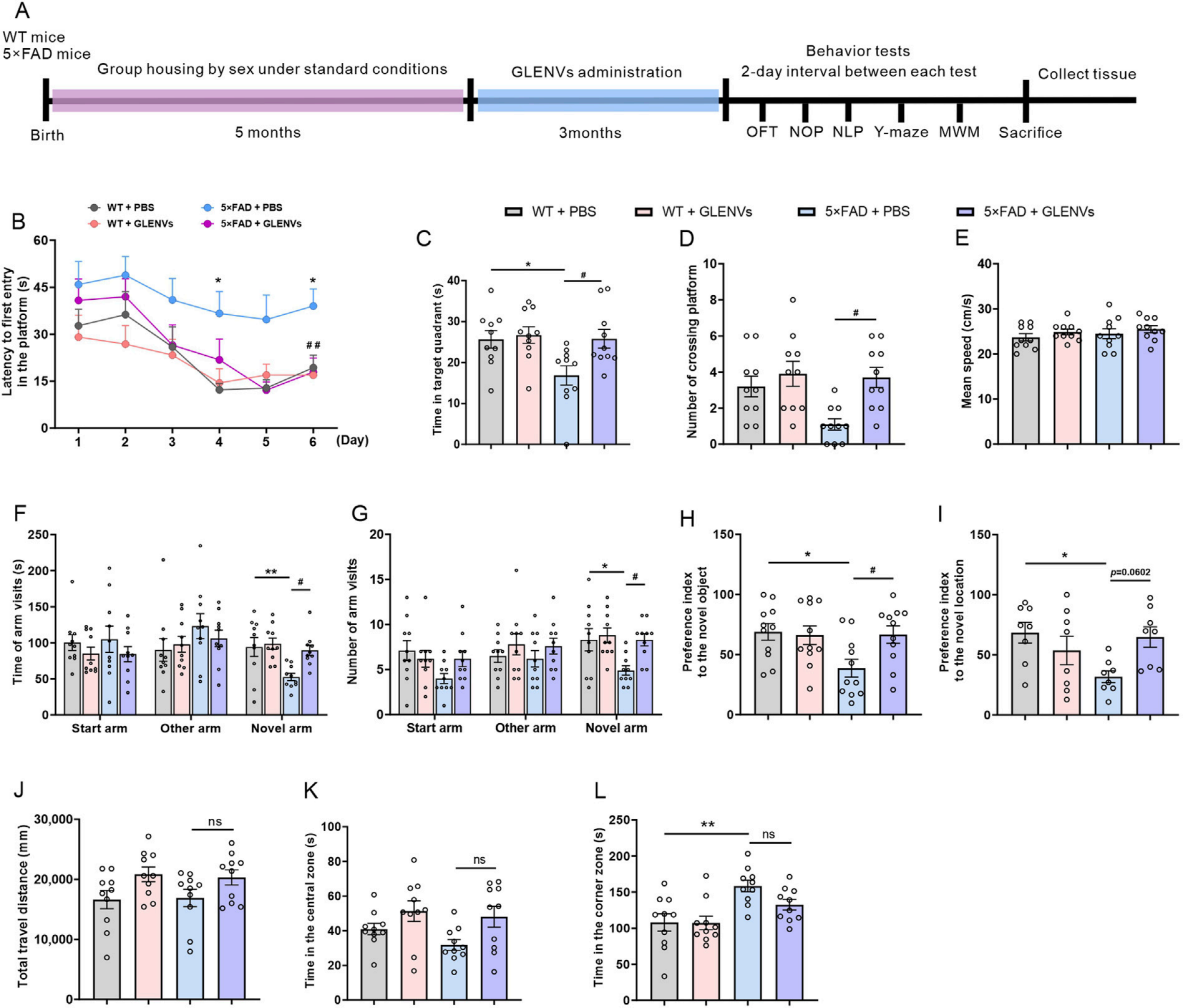
The alleviation of cognitive dysfunction in the 5×FAD mice by the treatment with GLENVs. **(A)** Schematic diagram of GLENVs treatment and behavioral tests. **(B)** The latencies to the daily first entry in the platform for the six consecutive days in the Morris water maze (MWM) test. **(C)** Searching time that the mice stayed in the target quadrant. **(D)** Crossings over the platform and **(E)** mean swimming speed of mice in the MWM test. **(F)** Time and **(G)** numbers of arm visits of mice in the Y-maze test. **(H)** Preference index to the novel object and **(I)** location in the novel object preference (NOP) and novel location preference (NLP) test. **(J)** Total distance, **(K)** time in the central zone, and **(L)** time in the corner zone of mice in the open field test (OFT). Data are expressed as mean ± SEM. n = 10 per group; *p < 0.05, **p < 0.01, compared with the WT + PBS group; #p < 0.05, compared with the 5×FAD + PBS group.

The experimental paradigm is depicted in Figure 2A. The locomotor activity and cognitive functions in mice were assessed by the OFT and subsequently by NOP, NLP, Y-maze test, and MWM test with a 2-day interval. In the MWM test, as illustrated in Figure 2B, the time required to locate the hidden platform (escape latency) was comparable among all groups on the first training day and became progressively shorter as the training days advanced. On the test day, the reduction in latency was statistically significant. Although no significant interaction was evident between genotypes and GLENVs, both exerted a main effect on escape latency [F (1, 36) = 8.223, p = 0.0069; F (1, 36) = 6.538, p = 0.0149, respectively]. Compared with the WT-mice, the 5×FAD mice exhibited memory impairment and took longer to locate the platform (p = 0.0361), which was markedly improved after GLENVs treatment (p < 0.0001) (Figure 2B). For the time spent in the target quadrant [F (1, 36) = 4.895, p = 0.0334; F (1, 36) = 5.160, p = 0.0292, respectively] (Figure 2C) and the crossings over of the platform region [F (1, 36) = 6.473, p = 0.0154; F (1, 36) = 6.473, p = 0.0154, respectively] (Figure 2D), no significant interaction between genotypes and GLENVs treatment was reported but both displayed significant main effects in all groups; *post hoc* analysis indicated that compared with t the control mice, the 5×FAD mice spent substantially shorter time in the target quadrant and crossed less over the platform region (p = 0.0361; p = 0.0494, respectively); and after the administration of GLENVs, the residence time of the 5×FAD significantly increased (p = 0.0326; p = 0.0494, respectively) when compared with their counterparts without GLENVs treatment. No marked difference in the swimming speed was evident among the mice in each group (Figure 2E).

In the Y-maze test, no significant interaction was evident between genotypes and GLENVs treatment, but both had a main effect on the total time of open arm entry [F (1, 36) = 8.334, p = 0.0065; F (1, 36) = 5.550, p = 0.0240] and the number of open arm entries [F (1, 36) = 5.251, p = 0.0279 for both] (Figures 2F,G). Compared with control animals, the 5×FAD mice exhibited reduced exploration time and fewer entries into the novel arms (p = 0.0098; p = 0.0368, respectively), which was noticeably improved after GLENVs treatment (p = 0.0256; p = 0.0368, respectively).

In the NOP and NLP test, as shown in Figures 2H,I, an interaction was found between genotype and GLENVs treatment [F (1, 36) = 4.504, p = 0.0408; F (1, 36) = 8.004, p = 0.0076]. Post-hoc analysis revealed that compared with the control mice, the 5×FAD mice spent significantly less time exploring the new object or new location (p = 0.0437; p = 0.0199, respectively), while after the administration of GLENVs, the 5×FAD mice dramatically increased the time exploring the novel object (p = 0.0469) but not the novel location.

In the OFT, no marked interaction between Genotypes and GLENVs treatment was detected in all groups when the total distance of spontaneous movements and the time spent in the central or corner zones were examined (Figures 2J–L). Further analyses disclosed no main effects of genotypes, a marked main effect of GLENVs treatment on the time spent in the central zone in the OFT [F (1, 36) = 7.631, p = 0.0090] (Figure 2K), and a significant main effect for genotype treatment on the time spent in the corner zone in the OFT [F (1, 36) = 16.42, p = 0.0003] (Figure 2L). Post-hoc analysis demonstrated that compared with the control mice, the 5×FAD mice spent substantially longer time in the corner (p = 0.0028), suggesting that the 5×FAD mice seem to prefer to stay in the corners of the arenas. Collectively, these findings evidence that GLENVs treatments can mitigate the cognitive impairment of 5×FAD mice.

To further evaluate the effects of GLENVs treatment on the sex of the mice, we analyzed the behavioral performance of the male and female mice. The analysis showed that GLENVs treatment greatly alleviated the cognitive impairments in both female and male mice across the behavioral tests. In the MWM test (Supplementary Figures S3A,B), both female and male mice spent more time in the target quadrant (p = 0.0451; p = 0.0474, respectively) and crossed more frequently over the platform (p = 0.0656; p = 0.0530, respectively); in the Y maze test (Supplementary Figures S3C,D), both female and male mice reported a longer exploration time (p = 0.0208; p = 0.0122, respectively) and more novel arm entries (p = 0.0312; p = 0.0175, respectively); in the NOR test (Supplementary Figure S3E), both female and male mice prolonged the time exploring the novel object (p = 0.0201; p = 0.0327, respectively). Between the female and male mice, no statistical difference was found across the behavioral tests.

### 3.5 GLENVs treatment ameliorates Aβ pathology in 5×FAD mice

**FIGURE 3:**
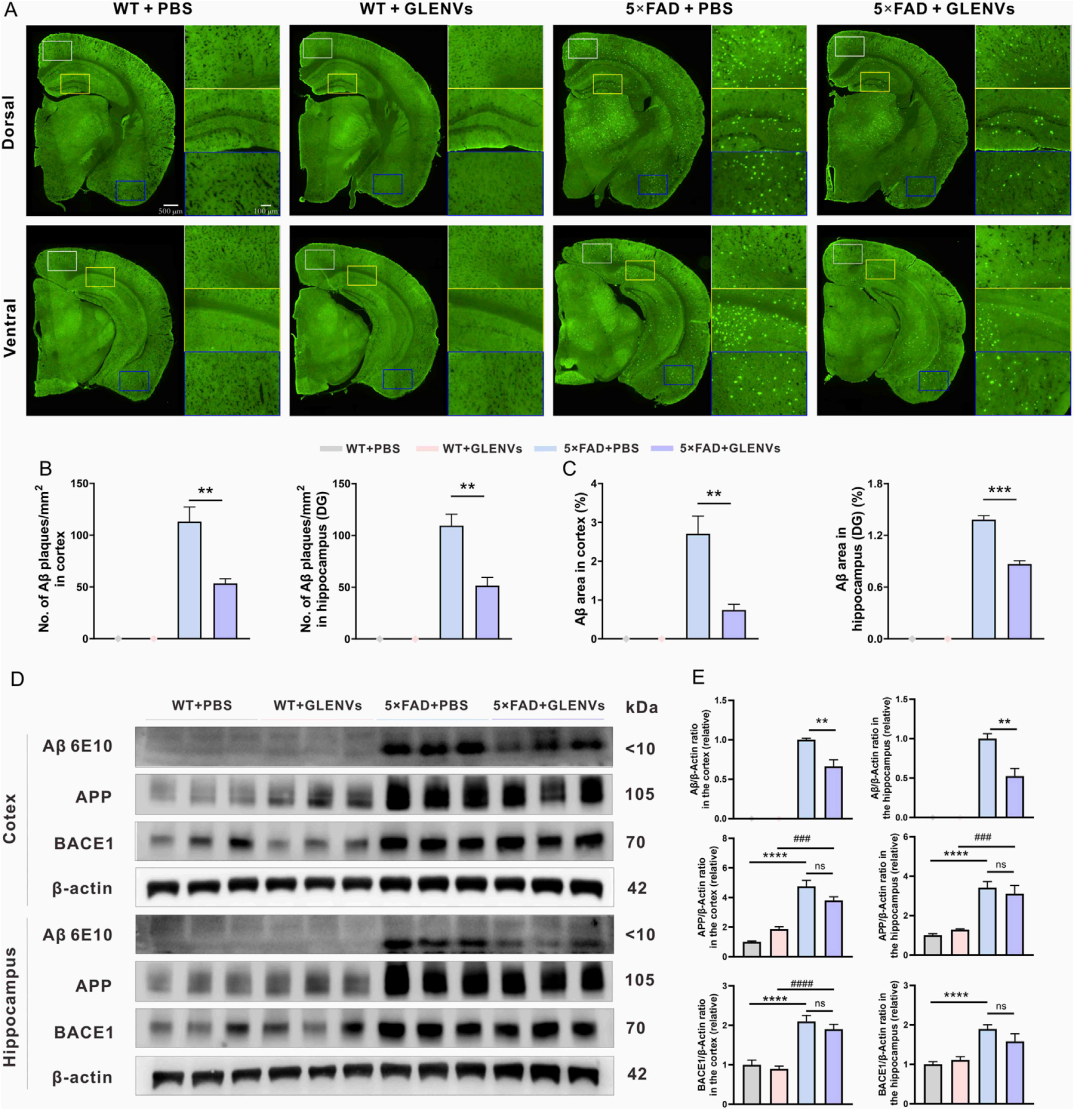
The amelioration of Aβ pathology by the treatment with GLENVs. **(A)** Representative images of TS staining in the brain sections of the 8-month-old WT and 5×FAD mice, and the enlarged images of cortex and hippocampal region. **(B)** Quantification of TS-positive Aβ plaques number and **(C)** area in the cortex (left) and hippocampus (right) of the 8-month-old WT and 5×FAD mice (n = 3 per group). **(D)** Western blotting analysis of Aβ, APP and BACE1 in the cortex or hippocampus of the 8-month-old WT and 5×FAD mice. **(E)** Relative protein level of Aβ, APP, BACE1 in the cortex and hippocampus (n = 5 mice per group). Data are expressed as mean ± SEM. **p < 0.01, ***p < 0.001, compared with the 5×FAD + PBS group; ^###^p < 0.001, ^####^p < 0.0001, compared with the 5×FAD + GLENVs group.

To investigate whether GLENVs treatment impacts the Aβ pathology in 5×FAD mice, TS staining was employed to assess Aβ plaques in brain sections of 5×FAD and wide-type mice. As shown in Figure 3A, plaques were scarcely detected in the WT mice, whereas denser Aβ plaques were observable in the cortex, dorsal and ventral hippocampus of the 5×FAD mice. However, after GLENVs treatment, the number of Aβ plaques and the Aβ area in the cortex and hippocampus of 5×FAD mice were significantly reduced (cortex: p = 0.0026, p = 0.0022; hippocampus: p = 0.0016, p < 0.0001, respectively) (Figures 3B,C). In line with the TS staining results, Western blotting results demonstrated that Aβ levels were markedly decreased in both the cortex (p = 0.047) and hippocampus (p = 0.0033) of the 5×FAD mice that received GLENVs treatmentwhen compared with their 5×FAD counterparts receiving no GLENVs administration (Figures 3D,E). Given that β-secretase 1 (BACE1) is a transmembrane protease that catalyzes the first step in the formation of amyloid β peptide from amyloid precursor protein (APP), no significant change in the expression of BACE1 and APP was evident after GLENVs treatment, indicating that GLENVs may not block the generation of Aβ (Figures 3D,E).

### 3.6 GLENVs treatment mitigates the inflammatory response in the 5×FAD mice

**FIGURE 4:**
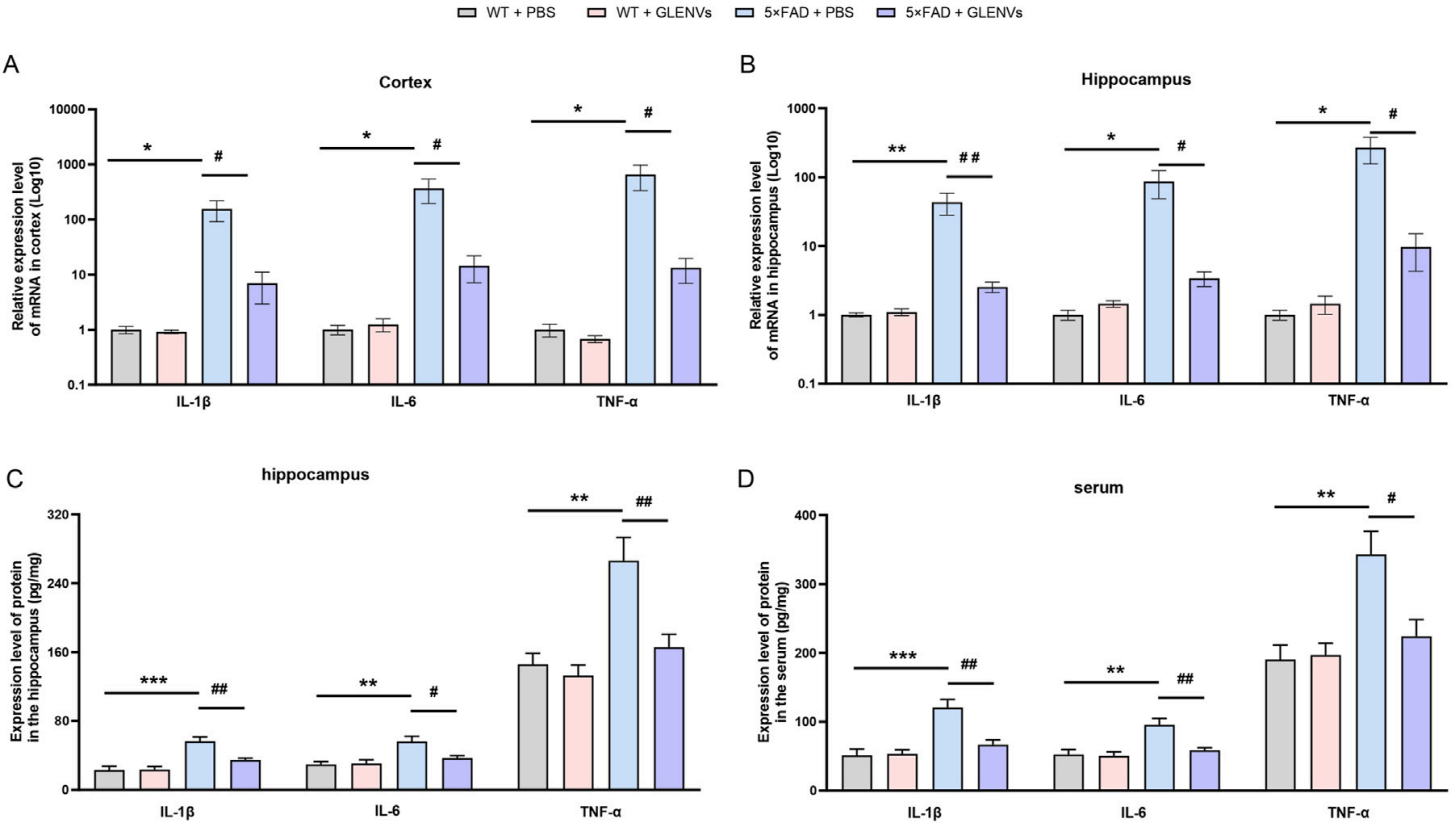
The GLENVs-induced reduction in the expression of inflammatory factors in serum, cortex and hippocampus of the 5×FAD mice. **(A and B)** The relative mRNA expression levels of IL-1β, IL-6 and TNF-α in the cortex **(A)** and hippocampus **(B)** (n = 6 per group). **(C and D)** The relative protein expression levels of IL-1β, IL-6 and TNF-α in the hippocampus **(C)** and serum **(D)** (n = 4 per group). Data are expressed as mean ± SEM. *p < 0.05, **p < 0.01, ***p < 0.001, compared with the WT + PBS group; #p < 0.05, ##p < 0.01, compared with the 5×FAD + PBS group.

To explore the role of GLENVs treatment in the 5×FAD mice, the level of the inflammatory factors IL-1β, IL-6 and TNFα was determined by qPCR and ELISA (Figure 4). In the cortex of mice (Figure 4A), an interaction between genotype and GLENVs treatment was noted in the mRNA levels of IL-1β [F (1, 20) = 5.377, *p* = 0.0311]. Genotype had a main effect on the levels of IL-1β [F (1, 20) = 6.298, *p* = 0.0208], IL-6 [F (1, 20) = 4.807, *p* = 0.0403] and TNF-α [F (1, 20) = 4.357, *p* = 0.0499]. The 5×FAD mice reported an increased expression of IL-1β (*p* = 0.0135), IL-6 (*p* = 0.0335) and TNF-α (*p* < 0.0409). After the 3-month GLENVs treatment, the level of inflammatory markers declined (IL-1β: *p* = 0.0180; IL-6: *p* = 0.0421; TNF-α: *p* = 0.0458).

In the hippocampus (Figures 4B,C), a marked interaction between genotype and GLENVs treatment was found on the mRNA (Figure 4B) and protein (Figure 4C) level of IL-1β [F (1, 20) = 7.119, *p* = 0.0148; F (1, 12) = 7.958, *p* = 0.0154], IL-6 [F (1, 20) = 4.843, *p* = 0.0397; F (1, 20) = 5.575, *p* = 0.0360, and TNF-α [F (1, 20) = 5.350, *p* = 0.0315; F (1, 20) = 6.109, *p* = 0.0294]. Both genotype and GLENVs treatment respectively had a main effect on the mRNA expressions of IL-1β [mRNA: F (1, 20) = 8.159, *p* = 0.0098; F (1, 20) = 7.048, *p* = 0.0152; protein: F (1, 12) = 32.27, *p* = 0.0001; F (1, 12) = 7.372, *p* = 0.0188], IL-6 [mRNA: F (1, 20) = 5.306, *p* = 0.0321; F (1, 20) = 4.740, *p* = 0.0416; protein: F (1, 12) = 14.71, *p* = 0.0024; F (1, 12) = 4.422, *p* = 0.0572] and TNF-α [mRNA: F (1, 20) = 6.053, *p* = 0.0231; F (1, 20) = 5.313, *p* = 0.0320; protein: F (1, 12) = 18.58, *p* = 0.0010; F (1, 12) = 10.17, *p* = 0.0078]. In the 5×FAD mice, an increase in mRNA and protein expressions of IL-1β, IL-6 and TNF-α was observed (IL-1β: *p* = 0.0045, *p* = 0.0003; IL-6: *p* = 0.0222, *p* = 0.0043; TNF-α: *p* = 0.0147, *p* = 0.0021); and the GLENVs treatment reduced the increased expressions of these cytokines (IL-1β: *p* = 0.0062, *p* = 0.0096; IL-6: *p* = 0.0269, *p* = 0.0361; TNF-α: *p* = 0.0186, *p* = 0.0082).

The expression levels of inflammatory factors in the peripheral blood serum were also detected. In the serum (Figure 4D), a significant interaction between genotype and GLENVs treatment was found on the level of the inflammatory factors [IL-1β: F (1, 12) = 10.20, p = 0.0077; IL-6: F (1, 12) = 6.595, p = 0.0246; TNF-α: F (1, 12) = 6.440, p = 0.0260]. Both genotype and GLENVs treatment respectively had a main effect on the expressions of IL-1β [F (1, 12) = 22.29, p = 0.0005; F (1, 12) = 8.939, p = 0.0113], IL-6 [F (1, 12) = 14.22, p = 0.0027; F (1, 12) = 8.079, p = 0.0148] and TNF-α [F (1, 12) = 13.31, p = 0.0033; F (1, 12) = 5.160, p = 0.0423]. Compared with the control group, the 5×FAD mice displayed, in the serum, a substantial increase in the mRNA level of IL-1β (p = 0.0006), IL-6 (p = 0.0036), TNFα (p = 0.0043), suggesting an enhanced inflammatory response. After the GLENVs treatment, the levels of all the markers were normalized (IL-1β: p = 0.0044; IL-6: p = 0.0112; TNF-α: p = 0.0082).

These data imply that extensive alterations in the level of pro-inflammatory factors may occur in the 5×FAD mice and that the treatment with GLENVs is effective in mitigating these levels.

### 3.7 GLENVs treatment reduces microglia and astrocyte activation and downregulates the JAK2/STAT3 signaling pathway in the hippocampus of the 5×FAD of mice

**FIGURE 5:**
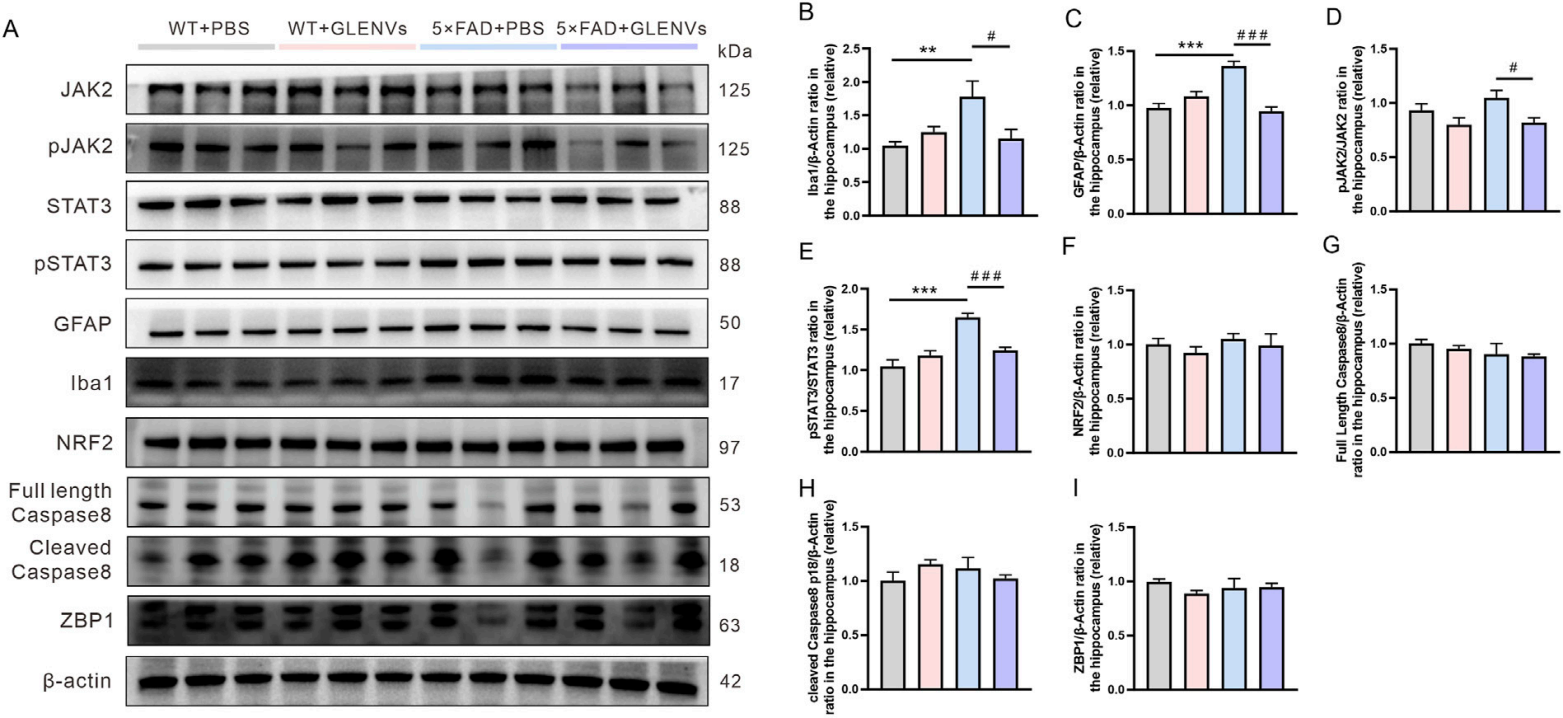
The GLENVs-induced inhibition of the activation of microglia and JAK2/STAT3 signaling pathway in the hippocampus of the 5×FAD mice. **(A)** Representative original Western blot bands showing expressions of Iba1, GFAP, JAK2, p-JAK2-Tyr1007, STAT3, p-STAT3-Tyr705, NRF2, Caspase 8 and ZBP1 in the hippocampus. **(B)** Relative protein level of Iba1. **(C)** Relative protein level of GFAP. **(D)** Relative protein level of p-JAK2-Tyr1007/JAK2. **(E)** Relative protein level of p-STAT3-Tyr705/STAT3. **(F)** Relative protein level of NRF2. **(G)** Relative protein level of full length Caspase 8. **(H)** Relative protein level of cleaved Caspase 8. **(I)** Relative protein level of ZBP1. Data are expressed as mean ± SEM. n = 7 per group; **p < 0.01, ***p < 0.001, compared with the WT + PBS group; #p < 0.05, ###p < 0.001, compared with the 5×FAD + PBS group.

Overactivated microglia and reactive astrocytes in the brain are pronounced features of AD (Hansen et al., 2018; Carter et al., 2019). The expression level of the biomarkers IBa1 and GFAP was detected respectively. In the hippocampus, a noticeable interaction between genotype and GLENVs treatment was observed in the level of IBa1 and GFAP [F (1, 24) = 8.078, p = 0.0090; F (1, 24) = 38.25, p < 0.0001] with genotype reporting a main effect on the expressions of IBa1 [F (1, 24) = 4.734, p = 0.0397] and GFAP [F (1, 24) = 8.645, p = 0.0071] (Figures 5A–C). Compared with the control group, the 5×FAD mice reported a marked increase in the protein level of IBa1 (p = 0.0083) and GFAP (p < 0.0001), indicating the activation of microglia and astrocytes. After GLENVs treatment, the levels of IBa1 and GFAP were drastically decreased (p = 0.0273; p < 0.0001, respectively).

As the JAK2/STAT3 signaling pathway, mainly comprising JAK2/p-JAK2-Tyr1007 and STAT3/p-STAT3-Tyr705, is a crucial inflammatory pathway in the brain (Chiba et al., 2009), we detected the expressions of JAK2, STAT3, p-JAK2-Tyr1007, and p-STAT3-Tyr705 by Western blotting. In the hippocampus, neither an interaction between genotype and GLENVs treatment nor a main effect of either was observed. However, compared with that of the control group, the phosphorylation of JAK2 at Tyr1007 decreased (Figures 5A,D). A marked interaction between genotype and GLENVs treatment was found in the level of the phosphorylation of STAT3 at Tyr705 [F (1, 24) = 21.03, p = 0.0001]; genotype and GLENVs treatment both had a main effect on the phosphorylation of STAT3 [F (1, 24) = 31.93, p < 0.0001; F (1, 24) = 5.119, p = 0.0330]. Compared with the control group, the 5×FAD mice reported, in the hippocampus, a drastic increase in the phosphorylation of STAT3 at Tyr705 (p < 0.0001). After GLENVs treatment, the phosphorylation of STAT3 was normalized ((p < 0.0001) (Figures 5A,E). No noticeable difference was observed in the total protein levels of JAK2 and STAT3 in the hippocampus among all groups. In addition, we also detected the expression levels of nuclear factor NRF2, a transcription factor that regulates oxidative stress, Caspase8 (a cell apoptosis initiator), and ZBP1, which regulates inflammation and apoptosis. The results showed no significant changes in the hippocampus after GLENVs treatment (Figures 5F–I).

### 3.8 GLENVs has no adverse effects on the functions of liver and kidneys

We examined the alterations in liver and kidney functions in the mice after GLENVs treatment (Tables 4 and 5). Hepatocellular injury often elevates serum AST and ALT, so the serum level of ALT and AST is the most sensitive and crucial marker of liver injury in clinical and laboratory settings. Blood urea nitrogen, serum creatinine, and uric acid, as critical indicators of renal function, are commonly used to diagnose impaired kidney function. The measurements of these biochemical indicators in mice are detailed in Tables 4 and 5, which revealed no significant changes in the levels of serum ALT, AST, blood urea nitrogen, creatinine, and uric acid in the four groups, indicating that GLENVs have no toxic effects on the liver and kidneys function of the mice.

**TABLE 4:** Effects of GLENVs on liver function indexes in mice (n = 6).

Group	Treatment	ALT (nmol/min/mL)	AST (nmol/min/mL)
Wide-type mice	PBS	44.69 ± 2.814	50.67 ± 5.026
	GLENVs	45.38 ± 5.365	49.83 ± 3.503
5×FAD mice	PBS	44.63 ± 5.213	50.01 ± 4.254
	GLENVs	43.58 ± 4.382	49.03 ± 5.045

**TABLE 5:** Effects of GLENVs on renal function indexes in mice (n = 6).

Group	Treatment	Creatinine (μmol/L)	Blood urea nitrogen (mmol/L)	Uric acid (μmol/L)
Wide-type mice	PBS	1,603 ± 63.71	1.902 ± 0.1306	689.8 ± 19.41
	GLENVs	1,489 ± 48.19	1.930 ± 0.0797	654.6 ± 28.08
5×FAD mice	PBS	1,535 ± 103.5	1.787 ± 0.1251	691.7 ± 46.21
	GLENVs	1,555 ± 100.2	1.718 ± 0.1223	672.2 ± 10.19

## 4 Discussion

**FIGURE 6:**
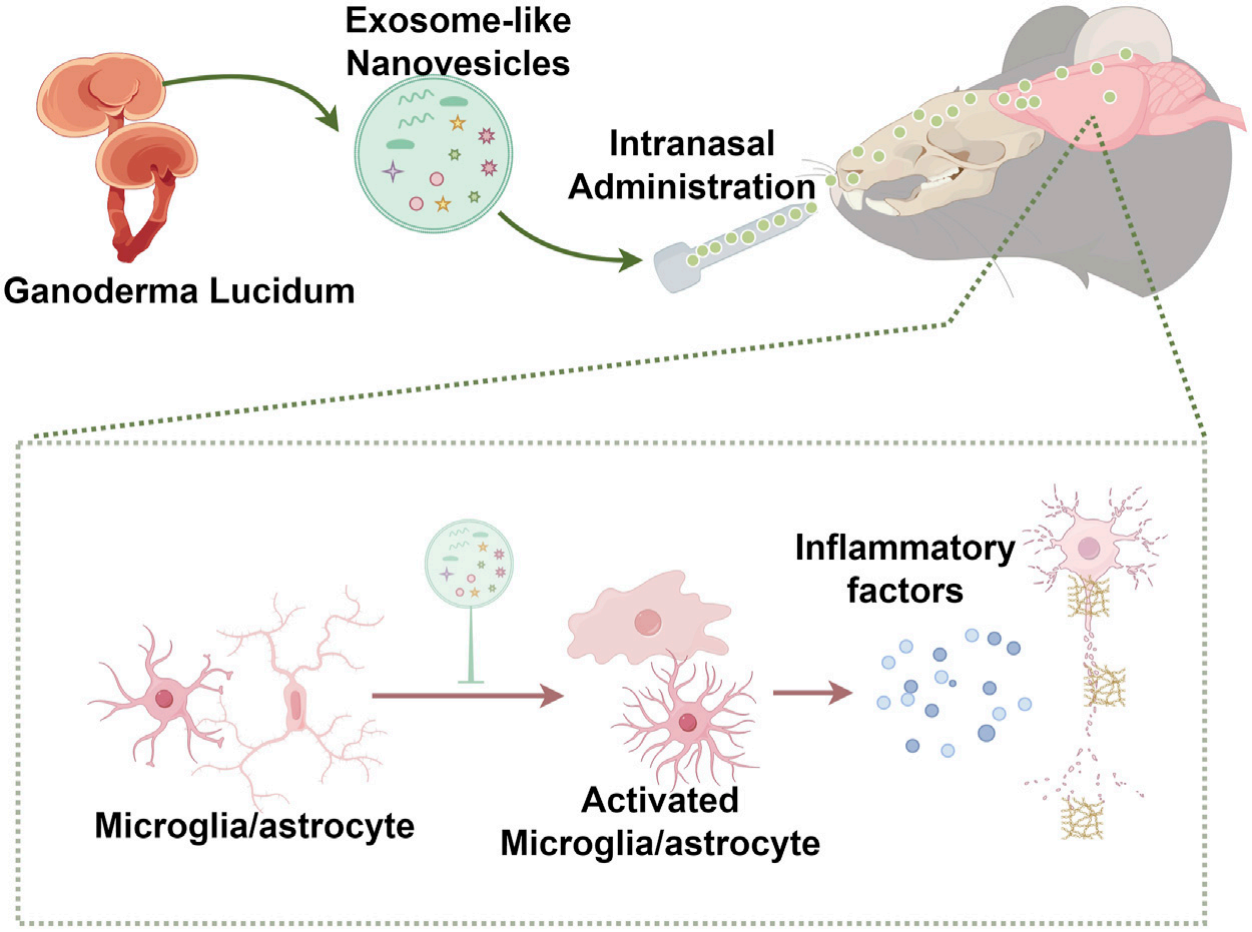
Scheme of the proposed mechanism.

In the current study, exosome-like nanovesicles from *G. lucidum* (GLENVs) were extracted by differential ultracentrifugation method and characterized. These GLENVs were administered to 5×FAD mice intranasally to verify their potential to cross the BBB and ameliorate the cognitive impairment in the AD mouse model. We found that these GLENVs successfully penetrated the BBB and attenuated the Aβ aggregation and inflammatory processes in the brain samples and effectively mitigated the behavioral abnormalities of the 5×FAD mice (Figure 6). The findings highlight that GLENVs may be potential therapeutic agents for AD treatments.

As a chronic disease, AD pathology may exist way before any symptoms are manifested during the mild cognitive impairment stage and ultimately progresses to overt dementia (Scheltens et al., 2021). Therefore, there has been an earnest call for early detection, early diagnosis and early treatment of the disease. The 5×FAD mice constitute a typical transgenic mouse model for AD research. In the current study, the intervention was initiated in the 5-month 5×FAD mice and followed by a 3-month treatment prior to behavioral tests, aiming to determine the benefits of GLENVs intervention in alleviating the disease when the pathological damage and behavioral abnormalities are not yet severe. This protocol is consistent with the clinical phenomenon that patients usually seek medical attention only when they encounter memory or learning difficulties. In our study, in comparison with the age-matched WT mice, the 8-month-old 5×FAD mice manifested impairment in cognition and spatial memory in multiple behavioral tests, which was significantly mitigated after the 3-month treatment of GLENVs. Further pathological analyses revealed that the Aβ plaque content and area were effectively controlled. Future systemic research is awaited to explore the regulatory effects of GLENVs on Aβ generation and clearance pathways.

*Ganoderma lucidum,* has been in use for thousands of years in the East Asia (Ahmad, 2018). As one of the principal bioactive and medicative constituents within *G. lucidum*, GLTs have attracted extensive attention in preclinical investigations. Remarkably, ganoderic acid A has been found to facilitate the Aβ clearance by promoting autophagy (Qi et al., 2021) or alleviate neuroinflammation (Zhang et al., 2021), thereby ameliorating cognitive impairments in AD mice. Additionally, deacetyl ganoderic acid F is found to attenuate lipopolysaccharide (LPS)-induced neural inflammation by inhibiting the activation of microglia and astrocytes (Sheng et al., 2019). Another bioactive component, *G. lucidum* polysaccharides, has been reported to enhance neurogenesis and alleviate cognitive impairment in transgenic mouse AD models (Huang et al., 2017). Although available literature suggests that *G. lucidum* may be a promising candidate for AD treatment, there is still a lack of clinical investigations regarding the efficacy of *G. lucidum* in treating AD patients. In a clinical trial involving 42 AD participants, the oral administration of *G. lucidum* spore powder for a 6-week period failed to improve cognitive performance in these patients, which may be related to the relatively short intervention duration or the difficulty the active components encounter in penetrating the BBB to exert their effects (Wang et al., 2018). Therefore, it is rewarding to explore safer and more cost-effective dosage forms of *G. lucidum.*

Recently, exosomes have become a prominent research focus. In view of the multiple advantages of exosomes, we endeavored to extract and successfully obtained them from *G. lucidum* by differential ultracentrifugation, which, to date, is deemed as the gold standard for exosome isolation and remains the most frequently utilized method for the purification of exosomes-like vesicles (Doyle and Wang, 2019). The process removes large-diameter and high-density components by gradually increasing the centrifugation speed and time, thus producing exosomes (Dad et al., 2021). So far, this method has successfully extracted a variety of exosomes from natural plants or large fungi, such as Shiitake Mushroom, momordica charantia, sunflower, edible ginger, turmeric, *etc.* (Liu et al., 2020; Cai et al., 2022; Regente et al., 2009; Zhang et al., 2016; Wang et al., 2024). To elucidate the composition of GLENVs, we adopted high-resolution mass spectrometry combined with metabolomics to identify the components within the vesicles. Unfortunately, due to the lack of *G. lucidum*-related information in the database, no active ingredients related to *G. lucidum* were identified. Given that the main active components of ginsenosides, including Rg1, Rg3, Re, and Rb1, can be detected in ginseng-derived exosome-like nanoparticle (Kim et al., 2023), a triple quadrupole mass spectrometer was employed to conduct qualitative and quantitative analysis of the important active components of *G. lucidum*. Fortunately, the selected ganoderic acids (ganoderic acids A, B, D, F, G, I, Lucidenic acid A and Ganoderenic acid D) were found in the GLENVs. We did not detect protein and RNA in GLENVs, mainly due to the scarcity of research into the types, content and functions of the proteins and RNAs in *G. lucidum*, which makes it infeasible to validate the function of GLENVs observed in our experiments from peer findings. The efficacy of GLENVs in treating 5×FAD mice suggests that further studies are awaited to explore the underlying mechanisms.

LC-QqQ MS/MS is an excellent tool for quantitative analysis of content at the picogram level. In this study, we adopted the LC-QqQ MS/MS approach to detect the active ingredients of ganoderic acid in the four batches. Among the eight selected known compounds in *G. lucidum*, ganoderic acid A exhibited the highest concentration, which was substantially higher than that of the other ganoderic acids, implying that it may play the most important role in pharmacological effects. As the marked structural similarity of ganoderic acids imposes considerable challenges on the purification process, the acquisition of standards is not easily accessible and there are only eight standards listed in this article. There may exist other ganoderic acids or other compounds within the GLENVs. Given the effectiveness of GLENVs, future research can further explore the component types and functions within GLENVs. In the content determination of the four batches, the fourth batch was slightly different from the first three batches, which may be due to the instability of extraction process or the variation in the proportion of active ingredients among different batches of *G. lucidum*. Therefore, future experiments may also investigate into the content determination of active ingredients in GLENVs at diverse growth periods and origins.

In this study, the intranasal administration was adopted for GLENVs treatment, for it bypasses the BBB and provides a direct drug delivery to the brain *via* the olfactory pathway, thereby increasing the bioavailability of drugs in the brain. Moreover, the encapsulation of drugs or active ingredients within exosomes reduces the clearance of drugs by the nasal mucosa and improves the delivery efficiency (Erdo et al., 2018; Passeri et al., 2022). LC-MS method was utilized to detect the content of ganoderic acids in the brain. Among the eight compounds tested, relatively high concentrations and robust MS response were found for ganoderic acids A, D and F, so they were selected as representative compounds to assess the BBB penetration of GLENVs. In our experiment, ganoderic acids A, D and F were detected in the olfactory bulb, cortex, hippocampus, hypothalamus tissue of the mice, despite some differences in the concentrations. The possible reasons may include insufficient mixing of GLENVs, inaccurate intranasal dosage, individual differences in the mice. Therefore, future research may need to involve more mice for testing.

Aβ deposition and neuroinflammation constitute the major pathological feature of AD (Su et al., 2016). Aβ deposition initiates the activation of microglia and astrocytes, and subsequently triggers the expression of various inflammatory and anti-inflammatory cytokines (Thakur et al., 2023). Research has revealed that many pro-inflammatory cytokines, including IL-6, IL-1β, and TNF-α, are significantly elevated in the peripheral blood of AD patients and that their levels in the brains of these patients are also increased (Licastro et al., 2000; Swardfager et al., 2010). These pro-inflammatory cytokines can induce neural damage and accelerate the progression of neurodegeneration in AD by promoting Aβ deposition, increasing the permeability of the BBB, and mediating cytotoxicity (Su et al., 2016; Eikelenboom et al., 2012). Thus, a vicious cycle seems to exist between inflammation and Aβ, exacerbating the progression of AD pathology. Consistent with this notion, in our study, when compared with the control mice, the 5×FAD mice reported a significant increase in the number and area of Aβ, the upregulation of IBA1 and GFAP, and the expression of pro-inflammatory cytokines. These findings highlight that neuroinflammation might be a potent driver of AD progression and that reducing neuroinflammation may be an essential part of the AD treatments. At present, studies have documented that TNF-α monoclonal antibodies can reduce Aβ plaque formation and alleviate the symptoms of AD (Shi et al., 2011) and that a long-term use of nonsteroidal anti-inflammatory drugs can inhibit AD progression (McGeer et al., 1996; Anthony et al., 2000), implying that a targeted reduction of neuroinflammation is indeed effective in alleviating the progression of AD. In our study, after GLENVs treatment, the 5×FAD mice reported a significant decrease in the number of Aβ plaques, the Aβ area, and the expression of IBA1 and GFAP, accompanied by a significant reduction in inflammatory factors, suggesting that the administration of GLENVs may attenuate the Aβ pathology and neuroinflammation in AD mice.

Janus kinase (JAK)/Signal transducer and activator of transcription (STAT) are involved in an important signaling pathway that regulates inflammation, oxidative stress and apoptosis in the brain. In AD pathology, this pathway mainly engages in the inflammatory regulation (Ni et al., 2023). JAK2, an important member of the JAK family, can be activated by IL-6, IL-1β, and TNFα at the Tyr1007 site. Phosphorylated JAK2 can promote the activation of STAT3 at the Tyr705 site and subsequently, the phosphorylated STAT3 initiates the transcription of target genes encoding pro-inflammatory factors and promotes the M1 polarization of microglia, ultimately leading to severer neuroinflammation (Nicolas et al., 2013). Recent studies have revealed that key proteins of the JAK2/STAT3 signaling pathway, including p-JAK2-Tyr1007 and p-STAT3-Tyr705, have been upregulated in the cortex and hippocampus of various AD mouse model (Ni et al., 2023; Long et al., 2021) and that the inhibition of JAK2 can attenuate the increase in inflammatory markers in microglia from APP/PS1 mice (Jones et al., 2015). Other studies have found that inhibiting the JAK2/STAT3 signaling pathways can alleviate microglia-mediated neuroinflammation injury and exert anti-inflammatory and neuroprotective effects (Song et al., 2016). In addition, the JAK/STAT pathway has been identified as an important pathway that regulates plaque deposition (Chintapaludi et al., 2020) and STAT3 plays an important role in the plaque clearance by microglia (Guillot-Sestier et al., 2015). Genomic and clinical data analyses also suggest that JAK-STAT signaling can serve as a therapeutic target for AD (Nevado-Holgado et al., 2019). In this study, the expression of p-STAT3-Tyr705 was significantly enhanced in the hippocampus of the 5×FAD mice when compared with the control group, while the expression of JAK2 and STAT3 did not change significantly. After the GLENVs treatment, a significant reduction was found in neuroinflammation and the expression of pJAK2-Tyr1007 and p-STAT3-Tyr705 in the hippocampus. Our findings indicate that GLENVs ameliorate neuroinflammation by inhibiting the JAK2/STAT3 signaling pathway, thereby conferring cognitive improvement in the 5×FAD mice.

Another study of a humanin derivative, which reports a complete restoration of cognitive function in an AD model, suggests that p-STAT3 levels in hippocampal neurons may decrease in an age-dependent manner in both AD mouse model and AD patients (Chiba T et al., 2009). This finding seems inconsistent with our current experimental results. The possible explanations are as follows: In the mouse experiment, the levels of pSTAT3 may vary in different AD mouse models, with the Tg2576 mice in the aforementioned article and the 5×FAD mice in our study; additionally, the levels of pSTAT3 may differ among mice of different ages. In the aforementioned article, the authors focused on the expression of pSTAT3 at the age of 12, 20, and 28 months. Although the expression of pSTAT3 in 6-month-old mice was also detected, no relevant statistical analysis was provided. The autopsied brain sections selected by the authors were from patients with sporadic AD around 75 years old, which is equivalent to approximately 22.5 months of age in mice (Dutta S and Sengupta, 2016). There is a substantial age disparity between them and the 8-month-old mice in our study, so the level of pSTAT3 might be different.

Compared with traditional extraction and purification methods, from an overall perspective, the use of GLENVs demonstrates greater advantages for to the treatment of nervous system diseases. GLENVs can be intranasally administered (Mondal et al., 2023), which can target and penetrate the BBB to directly exert therapeutic effects in the brain. In contrast, active ingredients of *G. lucidum* obtained through extraction and purification with organic solvents are mainly administered *via* oral gavage or intraperitoneal injection (Huang et al., 2017; Qi et al., 2021). Due to their relatively large molecular weight (typically exceeding 500 Da) and strong polarity, it is difficult for them to cross the BBB (Lipinski et al., 2001). When orally administered, the bioactive component of *G. lucidum* exhibits a low bioavailability (Cao FR et al., 2017; Zhang Q et al., 2009) and the desired therapeutic effect can only be warranted *via* a high dose or frequent administration, which brings on unwanted systemic side effects and raises penitential toxicity concerns. Notably, GLENVs preserve the natural combination of active molecules secreted by *G. lucidum*, including, but not limited to, active components, miRNA, functional proteins and lipid signaling molecules. These components are highly likely to exert a synergistic effect on the treatment and prevention of diseases. Compared with single specific components obtained by traditional methods, GLENVs better emulate the holistic biological effects of *G. lucidum* organism.

Some limitations remain in the current study. As GLENVs encompass a variety of’ *G. lucidum* active ingredients, it is possible that the alleviation of cognitive impairment is not solely attributable to the reduction of inflammation. Therefore, other mechanisms should be further explored in the future. In addition, in the current study, we did not detect components such as proteins, RNA and other secondary metabolites in GLENVs, which might potentially contribute to the pharmacological effects. *Ganoderma lucidum* has been widely accepted by many individuals as a health food and medicine. If GLENVs are to be developed into drugs in the future, the drug compliance will be relatively favorable. Extensive and in-depth research on aspects such as quality control, stability, content and mechanism, is essential to facilitate the clinical application of GLENVs, which is precisely the focus of our follow-up work. Given that AD is a chronic pathological progression, besides the exploration and development of novel drugs and formulations, multi-domain interventions, including a nutrition component with a low-sugar and low-fat diet (Scarmeas et al., 2018; Dilmore et al., 2023), increased intelligence training (Sitzer et al., 2006), as well as regular aerobic exercise (Brooks et al., 2024; Ryan and Kelly, 2016), might also hold some promise for the prevention of cognitive impairment.

## 5 Conclusion

The current study evidences that the intranasal administration of GLENVs can alleviate cognitive impairment in the 5×FAD mice by lessening neuroinflammation. These *G. lucidum* extracts produce no obvious toxicities on the liver and kidneys. These findings signify the therapeutic value of GLENVs in AD treatments.

## Data Availability

The original contributions presented in the study are included in the article/Supplementary Material, further inquiries can be directed to the corresponding authors.

## Glossary

Aβ: Amyloid beta
ALT: Alanine aminotransferase
ANOVA: Two-way analysis of variance
BBB: Blood-brain barrier
ELISA: Enzyme-linked Immunosorbent Assay
ESI: Electrospray ionization
FDA: Food and Drug Administration
*G. lucidum*: *Ganoderma lucidum*
GLENVs: *G. lucidum*-derived exosome-like nanovesicles
GLPs: *Ganoderma lucidum* polysaccharides
GLTs: *Ganoderma lucidum* triterpenoids
IL-1β: Interleukin-1β
IL-6: Interleukin-6
JAK: Janus kinase
JAK2: Janus kinase 2
LC-MS: Liquid chromatography - mass spectrometry
LC-QqQ-MS/MS: Triple quadrupole liquid chromatography tandem mass spectrometry
MRM: Multiple reaction monitoring
MSCs: Mesenchymal stem cells
MWM: Morris water maze
NLP: Novel location preference
NMDA: N-methyl-D-aspartic acid
NOP: Novel object preference
NTA: Nanoparticle tracking analysis
OFT: Open field test
PBS: Phosphate buffer solution
qRT-PCR: Quantitative Real-time PCR
SAST: Aspartate aminotransferase
STAT3: Signal transducer and activator of transcription
STAT3: Signal transducer and activator of transcription 3
TEM: Transmission Electron Microscopy
TNFα: Tumor necrosis factor-α
TS: Thioflavin S
WT: Wild-type
