# Supplementary material for: Intranasal administration of Ganoderma lucidum-derived exosome-like nanovesicles ameliorates cognitive impairment by reducing inflammation in a mouse model of Alzheimer’s disease
Source: Front Pharmacol. 2025 Jul 4;16:1572771. doi: 10.3389/fphar.2025.1572771 (PMC12271231; doi:10.3389/fphar.2025.1572771)
Supplement: Supplementary file 1 [file DataSheet1.docx]

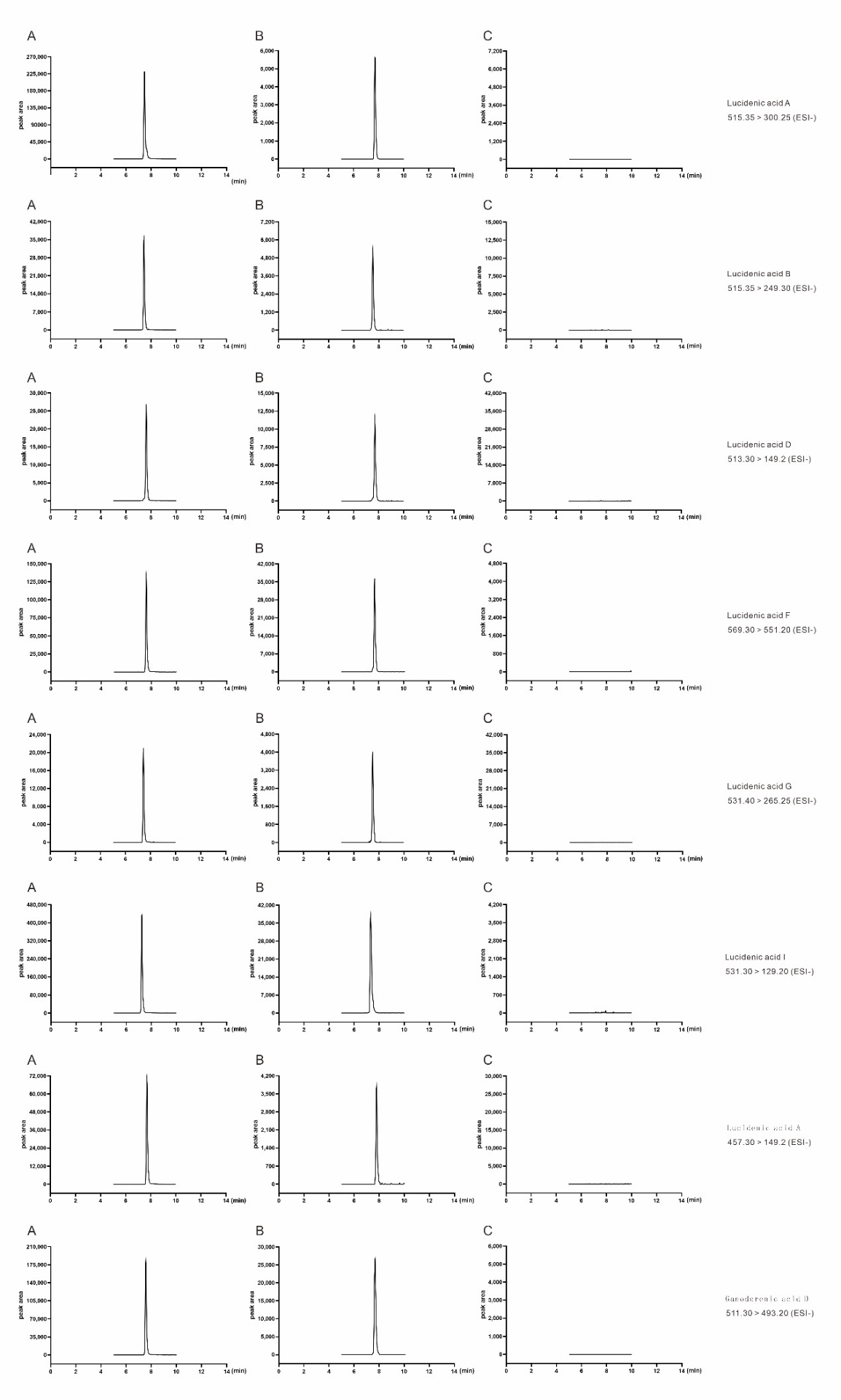


Figure S1. Representative extract ion chromatograms of the eight analytes. (A) standard substance; (B) GLENVs; (C) blank

**
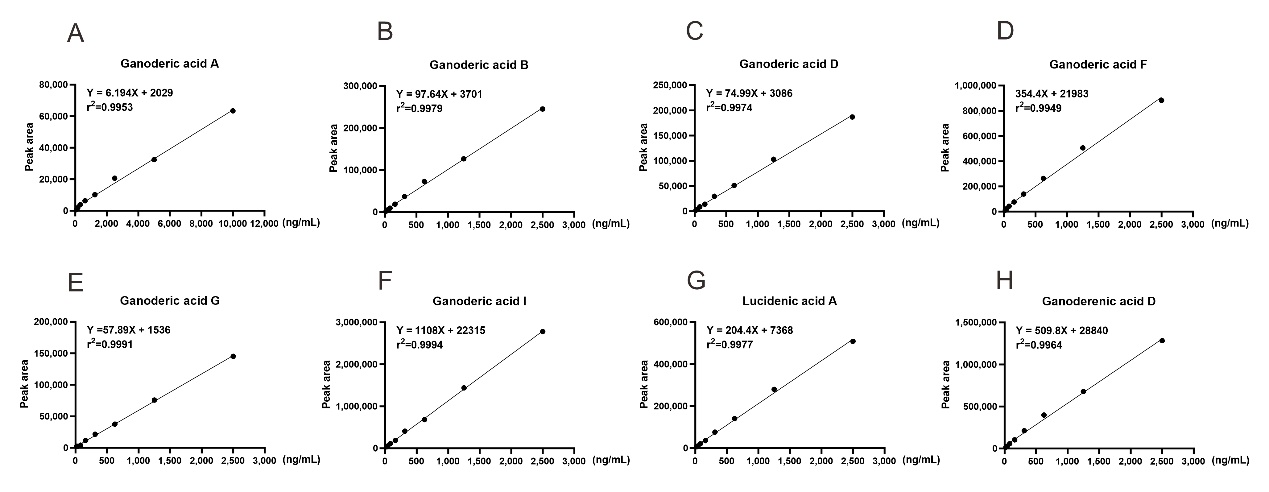
**

Figure S2. Standard curve and its corresponding standard equation of the eight analytes. (A) Ganoderic acid A. (B) Ganoderic acid B. (C) Ganoderic acid D. (D) Ganoderic acid F. (E) Ganoderic acid G. (F) Ganoderic acid I. (G) Lucidenic acid A. (H) Ganoderenic acid D.


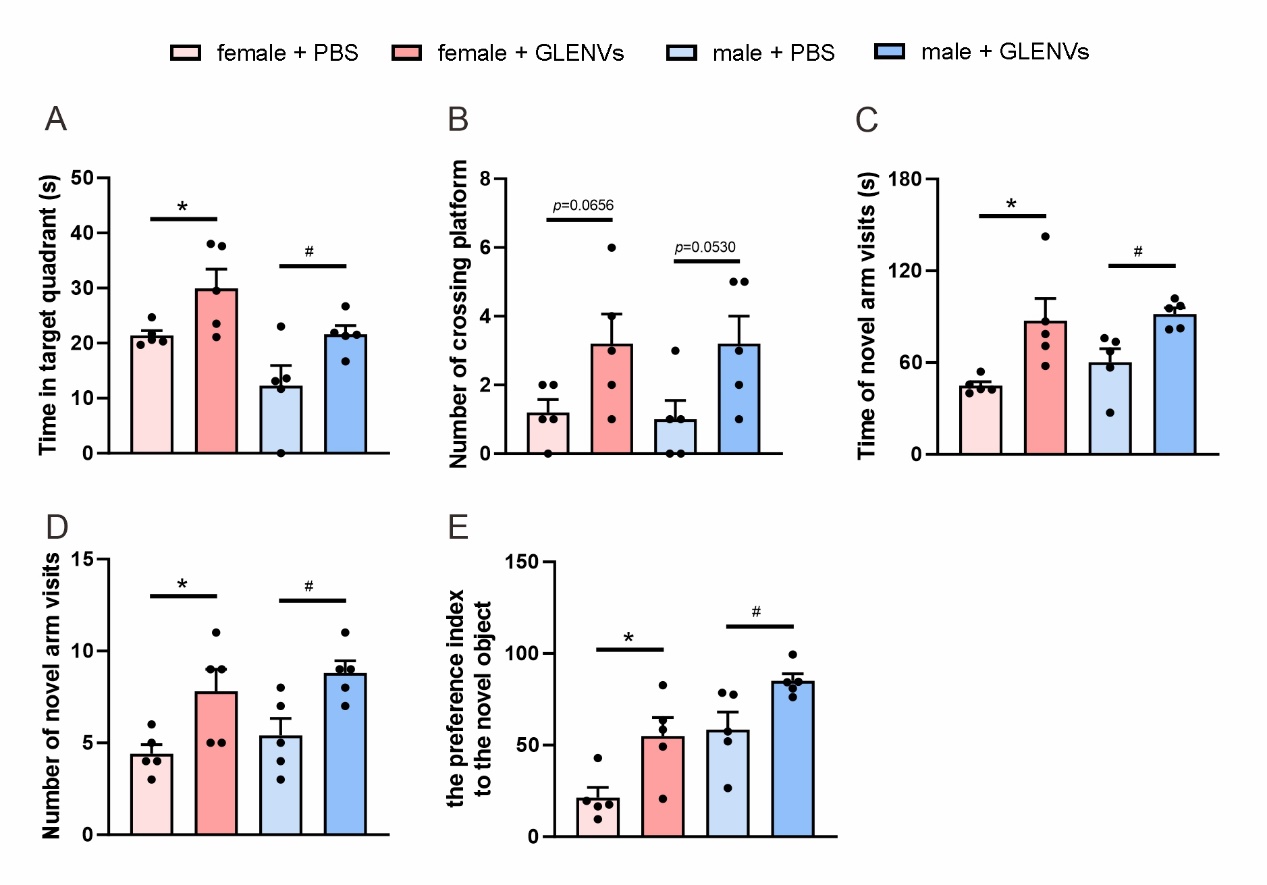


Figure S3. The alleviation in cognitive impairment in female and male 5×FAD mice by GLENVs. (A) Searching time in the target quadrant and (B) number of crossings over the platform of male and female mice in the MWM test. (C) Time and (D) number of arm visits of male and female mice in the Y-maze test. (E) Preference index to the novel object of male and female mice in the NOP test. Data are expressed as mean ± SEM. n = 5 per group; * p < 0.05, compared with the female + PBS group; # p < 0.05, compared with the male + PBS group. No statistical difference was found between the female and male mice across the tests.
